# Marine unsaturated fatty acids: structures, bioactivities, biosynthesis and benefits

**DOI:** 10.1039/c9ra08119d

**Published:** 2019-10-31

**Authors:** Yingfang Lu, Yinning Chen, Yulin Wu, Huili Hao, Wenjing Liang, Jun Liu, Riming Huang

**Affiliations:** Guangdong Provincial Key Laboratory of Food Quality and Safety, College of Food Science, South China Agricultural University Guangzhou 510642 China luyingfang@stu.scau.edu.cn DanielWu@stu.scau.edu.cn hhlhaohuili@stu.scau.edu.cn huangriming@scau.edu.cn +86 20 8528 3448; Guangdong Polytechnic College 526100 Zhaoqing China Yinning_Chen@163.com; Longgang No. 2 Vocational School Shenzhen 518104 China 345589207@qq.com; Laboratory of Pathogenic Biology, Guangdong Medical University Zhanjiang 524023 China lj2388240@gdmu.edu.cn +86 7592388240

## Abstract

Unsaturated fatty acids (UFAs) are an important category of monounsaturated and polyunsaturated fatty acids with nutritional properties. These secondary metabolites have been obtained from multitudinous natural resources, including marine organisms. Because of the increasing numerous biological importance of these marine derived molecules, this review covers 147 marine originated UFAs reported from 1978 to 2018. The review will focus on the structural characterizations, biological properties, proposed biosynthetic processes, and healthy benefits mediated by gut microbiota of these marine naturally originated UFAs.

## Abstract 2

Unsaturated fatty acids (UFAs) are an important category of monounsaturated and polyunsaturated fatty acids with nutritional properties.

## Graphical abstract

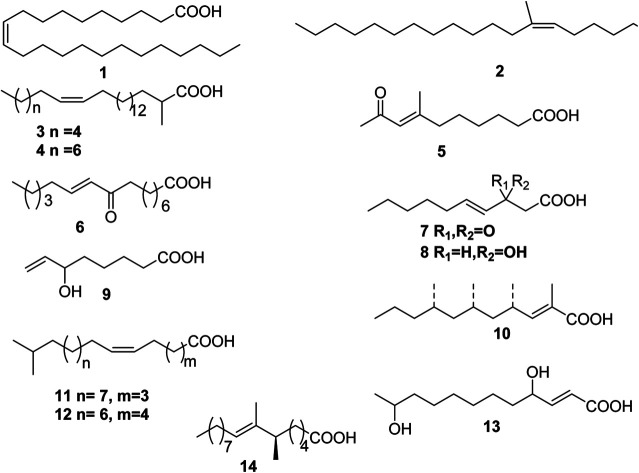

## 1 Introduction

Fatty acids other than saturated fatty acids (fatty acids that do not contain double bonds are called saturated fatty acids, and all animal oils, except fish oils, contain saturated fatty acids) are unsaturated fatty acids. Unsaturated fatty acids are a kind of fatty acid that makes up body fat. Unsaturated fatty acids (UFAs) consist of a long-chain hydrocarbon with the presence of at least one double covalent bond and ending in a carboxyl group (–COOH), and are distinguished into monounsaturated fatty acids and polyunsaturated fatty acids, both of which have numerous beneficial properties to human health.^1,2^ These secondary metabolites have previously been obtained from a variety of natural resources, including marine fish oils that are a good natural source of these UFAs.^3,4^ In previous decades, marine derived UFAs have attracted a great deal of interest because of their structural diversity and potential biological and nutritional functions.^5^ In particular, research interest in omega-3 fatty acids,^6^ eicosapentaenoic acid (EPA) and docosahexaenoic acid (DHA) from marine organisms, has dramatically increased as they are excellent sources of nutrients. These UFAs also can be described as *cis* fatty acids *versus trans* fatty acids, which is a description of the geometry of their double bonds. These characteristics in UFAs not only enable them to show a broad range of biological activities, but also allow the development of the nutrient-like physicochemical properties. However, most of marine derived UFAs belong to a relatively unexplored category that may hold a great promise for the potential nutritional application in the future. The structures and potential nutritional applications of UFAs, particularly these with the interesting biological activities have previously been reviewed,^7,8^ but there is still lack of a comprehensive review about marine derived UFAs. Thus, this review aims to summarize 147 marine organisms-derived UFAs published from 1978 to 2018. The review will focus on the structural characterizations, biological properties, proposed biosynthetic processes, and benefits mediated by gut microbiota of these marine UFAs. In addition, the origin of the isolation of these UFAs is also taxonomically presented.

## 2 Monounsaturated fatty acids

**Fig. 1:**
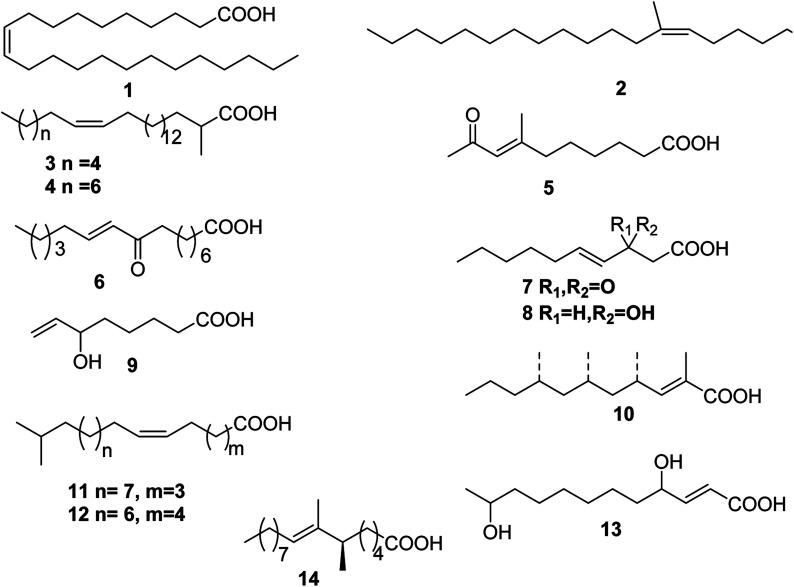
Structures of monounsaturated fatty acids from marine organisms.

Up to date, there are 14 of total monounsaturated fatty acids obtained from marine organisms, linear and branched monounsaturated fatty acids 1–14 (Table 1 and Fig. 1).

**Table 1:** Monounsaturated fatty acids from marine organisms

Number	Names	Bioactivities	Sources	Reference(s)
1	10-Tricosenoic acid	—	*Calyx podatypa*	9
2	(6*Z*)-7-Methyloctadec-6-enoic acid A	—	*Holothuria mexicana*	10
3	Not given	—	*Halichondria panicea*	11
4	Not given	—	*H. panicea*	11
5	Not given	—	*Ircinia* sp.	12
6	Not given	Antiinflammatory properties	*Gracilaria verrucosa*	13
7	Not given	—	*Ulva fasciata*	14
8	Not given	—	*U. fasciata*	14
9	Not given	—	*U. fasciata*	14
10	(2*E*,4*S*,6*S*,8*S*)-2,4,6,8-Tetramethyl-2-undecenoic acid	—	*Siphonaria capensis*	15
11	Not given	—	*S. denticulata*	16
12	Not given	—	*S. denticulata*	16
13	Seco-patulolide	—	*unidentified fungal strain*	17
14	Not given	—	*Sinularia* sp.	18

### 2.1 Linear monounsaturated fatty acids

#### 2.1.1 Sponges

Only one linear monounsaturated fatty acid, namely, 10-tricosenoic acid 1 was isolated from *Calyx podatypa*.^9^

### 2.2 Branched monounsaturated fatty acids

#### 2.2.1 Sea cubumber

The Caribbean sea cucumber *Holothuria mexicana* contained (6*Z*)-7-methyloctadec-6-enoic acid 2 that was found in the phospholipid fraction.^10^

#### 2.2.2 Sponges

Two long 2-methyl substituted fatty acids 3 and 4 were isolated as methyl esters from *Halichondria panicea* (Sea of Japan, Russia).^11^ 7-Methyl-9-oxo-dec-7-enoic acid 5 was isolated from an *Ircinia* sp. (Red Sea).^12^

#### 2.2.3 Algae

An extract with antiinflammatory properties from *Gracilaria verrucosa* (Jeju Is., S. Korea) yielded a keto fatty acid 6.^13^ A bioactivity-directed analysis of *Ulva fasciata* (Aabu-Qir, Mediterranean coast, Egypt) characterized three unsaturated fatty acids 7–9.^14^

#### 2.2.4 Limpets

(2*E*,4*S*,6*S*,8*S*)-2,4,6,8-Tetramethyl-2-undecenoic acid 10 was obtained from the South African pulmonate mollusc *Siphonaria capensis*.^15^ Two fatty acids 11 and 12 were isolated from the siphonarid limpet *Siphonaria denticulata*. The structures were confirmed by synthesis.^16^

#### 2.2.5 Microorganisms

An unidentified fungal strain (I96S215), which was obtained from a tissue sample of an unidentified marine sponge collected in Indonesia, produced seco-patulolide 13.^17^

#### 2.2.6 Corals

The absolute configuration of a unsaturated fatty acid 14, isolated from *Sinularia* sp. (Ishigaki Is., Okinawa), was determined by the Ohrui–Akasaka method.^18^

## 3 Polyunsaturated fatty acids

### 3.1 Linear chain polyunsaturated fatty acids

**Fig. 2:**
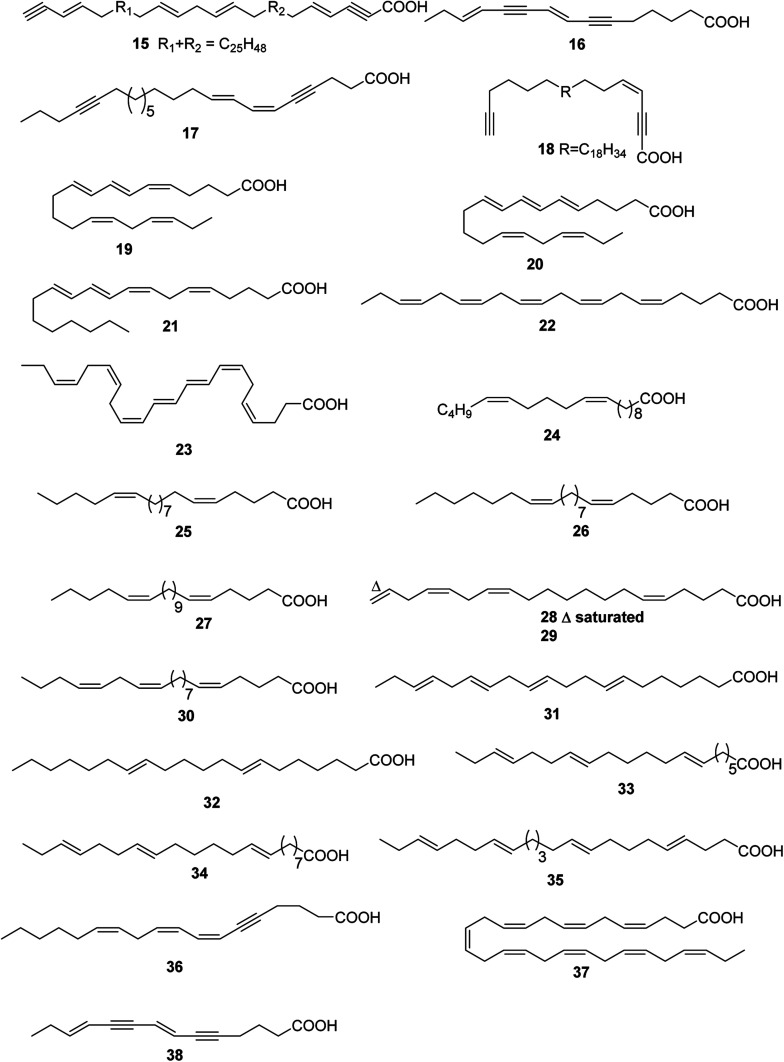
Structures of linear chain polyunsaturated fatty acids from marine organisms.

Up to date, there are 24 of total linear chain polyunsaturated fatty acids 15–38 obtained from marine organisms (Table 2 and Fig. 2).

**Table 2:** Linear polyunsaturated fatty acids from marine organisms

Number	Names	Bioactivities	Sources	Reference(s)
15	Not given	—	*Petrosia ficiformis*	19
16	Not given	Antimicrobial	*Oceanapia* sp.	20
17	Carduusyne A	—	*Phakellia carduus*	21 and 22
18	Petroformynic acid	—	*P. ficiformis*	23
19	(5*Z*,7*E*,9*E*,14*Z*,17*Z*)-Icosa-5,7,9,14,17-pentaenoic acid	—	*Ptilota jilicina*	24
20	(5*E*,7*E*,9*E*,14*Z*,17*Z*)-Icosa-5,7,9,14,17-pentaenoicacid	—	*P. jilicina*	24
21	5(*Z*),8(*Z*),10(*E*),12(*E*),14(*Z*)-Eicosapentaenoic acid	—	*Bossiella orbigniana*	25
22	(5*Z*,8*Z*,11*Z*,14*Z*,17*Z*)-Eicosapentaenoic acid	Inhibiting growth of the green alga *Monostroma oxyspermum*	*Neodilsea yendoana*	26
23	(4*Z*,7*Z*,9*E*,11*E*,13*Z*,16*Z*,19*Z*)-Docosaheptaenoic acid	—	*Anadyomene stellata*	27
24	10,15-Eicosadienoic acid	—	*Haminaea templadoi*	28 and 29
25	(5*Z*,15*Z*)-5,15-Eicosadienoic acid	—	*Calyptogena phaseoliformis*	30
26	(5*Z*,14*Z*)-5,14-Heneicosadienoic acid	—	*C. phaseoliformis*	30
27	(5*Z*,16*Z*)-5,16-Heneicosadienoic acid	—	*C. phaseoliformis*	30
28	(5*Z*,13*Z*,16*Z*)-5,13,16-Eicosatrienoic acid	—	*C. phaseoliformis*	30
29	(5*Z*,13*Z*,16*Z*)-5,13,16,19-Eicosatetraenoic acid	—	*C. phaseoliformis*	30
30	(5*Z*,14*Z*,17*Z*)-5,14,17-Heneicosatrienoic acid	—	*C. phaseoliformis*	30
31	7,11,14,17-Eicosatetraenoic acid	Anti-inflammatory	*Perna canaliculus*	31
32	7,13-Eicosadienoic acid	—	*Ophiura sarsi*	32
33	7,13,17-Eicosatrienoic acid	—	*O. sarsi*	32
34	9,15,19-Docosatrienoic acid	—	*O. sarsi*	32
35	4,9,15,19-Docosatetraenoic acid	—	*O. sarsi*	32
36	(7*Z*,9*Z*,12*Z*)-Octadeca-7,9,12-trien-5-ynoic acid	—	*Liagora farinosa*	33
37	4,7,10,13,16,19,22,25-Octacosaoctaenoic acid	—	Marine dinoflagellate species	33
38	7,11-Tetradecadiene-5,9-diynoic acid	—	Marine dinoflagellate species	33

#### 3.1.1 Sponges

One polyacetylene 15 was isolated from *Petrosia ficiformis*, but, as in several earlier examples, the structure was only partially elucidated.^19^ The antimicrobial constituent of a Japanese *Oceanapia* sp. was identified as the bis-acetylene 16.^20^ One acetylenic acid, carduusyne A 17, identified as the corresponding ethyl ester, was obtained from a specimen of *Phakellia carduus* obtained from a depth of 350 m by trawling.^21^ The compound 17 has been confirmed by a stereocontrolled synthesis.^22^ One additional polyacetylene, petroformynic acid 18, was isolated from both Atlantic and Mediterranean specimens of *Petrosia ficiformis*.^23^

#### 3.1.2 Algae

The temperate red alga *Ptilota jilicina* contained (5*Z*,7*E*,9*E*,14*Z*,17*Z*)-icosa-5,7,9,14,17-pentaenoic acid 19 and (5*E*,7*E*,9*E*,14*Z*,17*Z*)-icosa-5,7,9,14,17-pentaenoicacid 20, both of which were isolated as the corresponding methyl esters.^24^ Aqueous extracts of *Bossiella orbigniana* catalyse the enzymatic oxidation of arachidonic acid to bosseopentaenoic acid, 5(*Z*),8(*Z*),10(*E*),12(*E*),14(*Z*)-eicosapentaenoic acid 21, which was isolated from extracts of the alga.^25^ An allelopathic substance from *Neodilsea yendoana* that inhibited growth of the green alga *Monostroma oxyspermum* was identified as (5*Z*,8*Z*,11*Z*,14*Z*,17*Z*)-eicosapentaenoic acid 22.^26^ A polyunsaturated fatty acid, (4*Z*,7*Z*,9*E*,11*E*,13*Z*,16*Z*,19*Z*)-docosaheptaenoic acid 23, was encountered in *Anadyomene stellata* from Florida.^27^

#### 3.1.3 Mollusc

The eicosanoid 24, which was isolated from *Haminaea templadoi*,^28^ was synthesized in five steps.^29^ A series of n-4 polyunsaturated fatty acids including 25–30 were reported from the deep-sea clam *Calyptogena phaseoliformis* (Japan Trench).^30^ A homologous series of ω-3 polyunsaturated fatty acids, with 7,11,14,17-eicosatetraenoic acid 31 dominating, were isolated as anti-inflammatory components of the green-lipped mussel *Perna canaliculus* (New Zealand).^31^

#### 3.1.4 Echinoderm

Four nonmethylene interrupted polyunsaturated fatty acid derivatives 32–35 were identified in extracts of the brittle star *Ophiura sarsi*.^32^

#### 3.1.5 Others

Among the lipids of *Liagora farinosa* were four compounds that can be differentiated by UV absorption and/or the presence of an acetylene functionality. The metabolite, (7*Z*,9*Z*,12*Z*)-octadeca-7,9,12-trien-5-ynoic acid 36, was ichthyotoxic.^33^ Two very long, highly unsaturated fatty acids 37 and 38 were isolated from seven marine dinoflagellate species.^34^

### 3.2 Branched chain polyunsaturated fatty acids

**Fig. 3:**
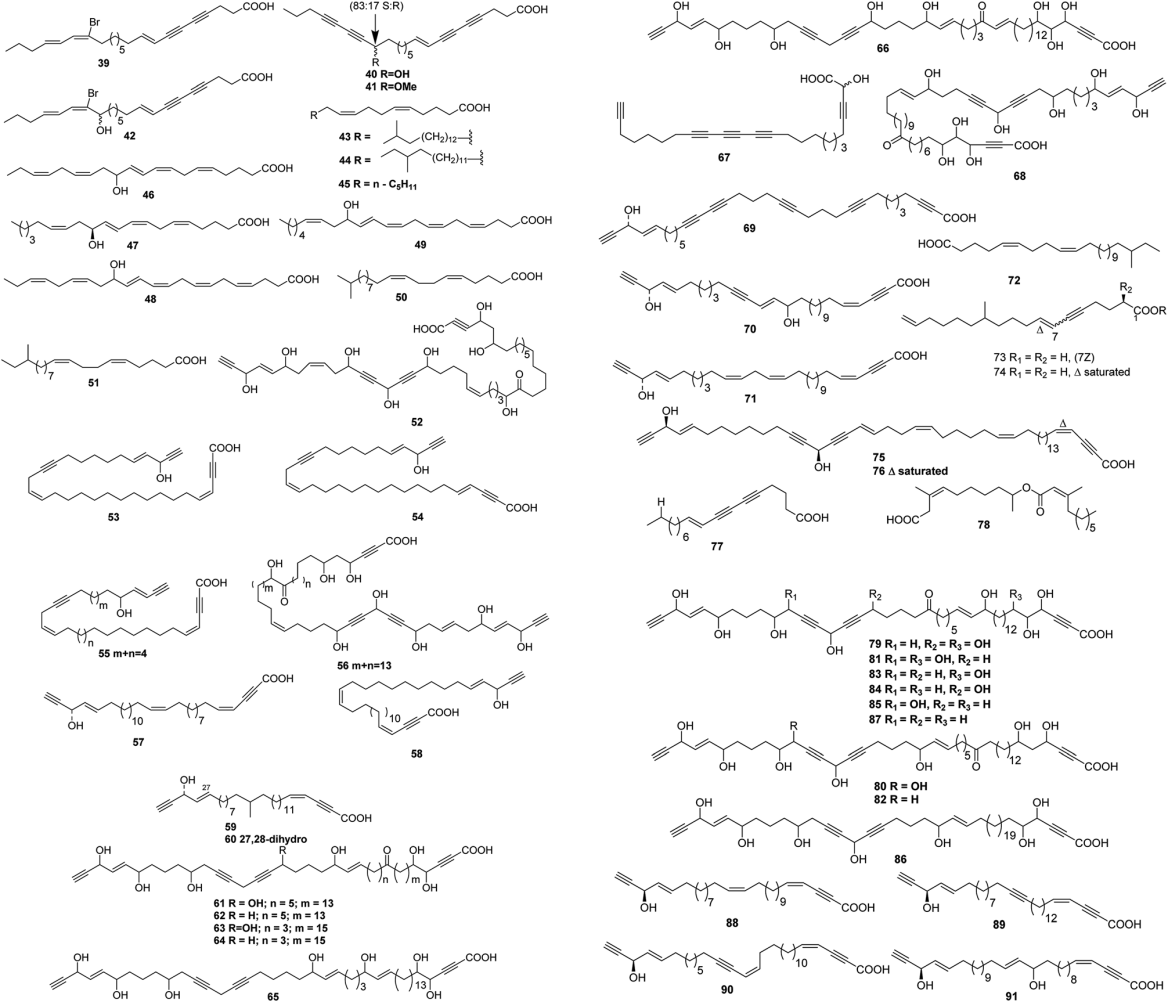
Structures of branched chain polyunsaturated fatty acids from sponges.

**Fig. 4:**
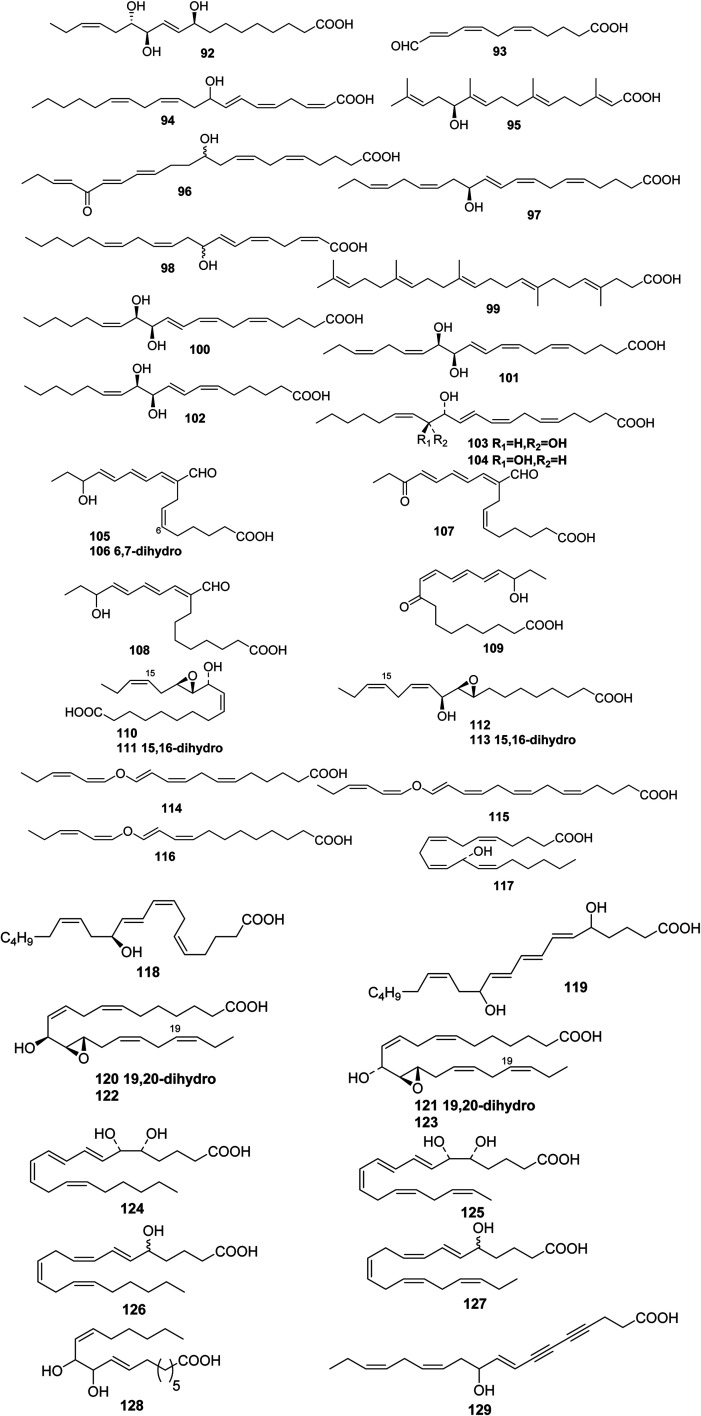
Structures of branched chain polyunsaturated fatty acids from marine algae.

**Fig. 5:**
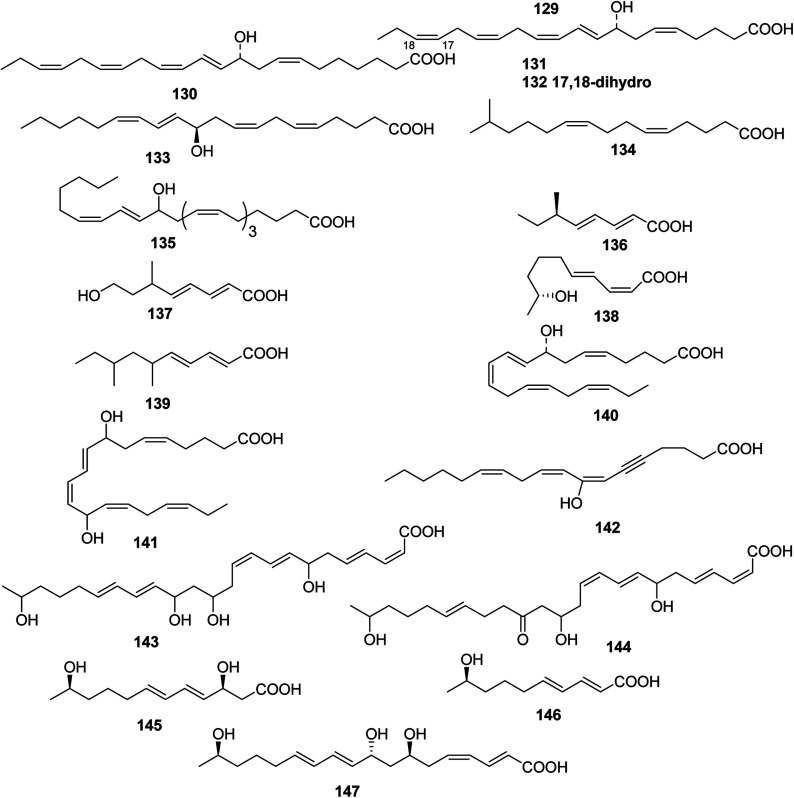
Structures of branched chain polyunsaturated fatty acids from Coelenterate, Marine fungus, Arthropoda, Bacterium.

Up to date, there are 109 of total linear chain polyunsaturated fatty acids 39–147 obtained from marine organisms (Tables 3–5 and Fig. 3–5).

**Table 3:** Branched chain polyunsaturated fatty acids from sponges

Number	Names	Bioactivities	Sources	Reference(s)
39	Not given	—	*P. carduus*	21
40	Not given	—	*P. carduus*	21
41	Not given	—	*P. carduus*	21
42	Not given	—	*P. carduus*	21
43	(*Z*,*Z*)-25-Methyl-5,9-hexacosadienoic acid	—	*Jaspis stellifera*	35
44	(*Z*,*Z*)-24-Methyl-5,9-hexacosadienoic acid	—	*J. stellifera*	35
45	(5*Z*,9*Z*)-Hexadeca-5,9-dienoic acid	—	*Chondrilla nucula*	36
46	5,8,10,14,17-Eicosapentaenoic acid	—	*Echinochalina mollis*	37
47	Not given	—	*E. mollis*	37
48	4,7,10,12,16,19-Docosahexaenoic acid	—	*E. mollis*	37
49	Not given	—	*E. mollis*	37
50	5,9-Eicosadienoic acid	—	*Erylus forrnosus*	38 and 39
51	5,9-Eicosadienoic acid	—	*E. forrnosus*	38 and 39
52	Petrosolic acid	Inhibited HIV reverse transcriptase	*Petrosia* sp.	40
53	Corticatic acid A	Antifungal	*Petrosia corticata*	41
54	Corticatic acid B	Antifungal	*P. corticata*	41
55	Corticatic acid C	Antifungal	*P. corticata*	41
56	Nepheliosyne A	—	*Xestospon*	42
57	Triangulynic acid	Against leukemia and colon tumour lines	*Pellina triangulata*	43
58	Pellynic acid	Inhibited inosine monophosphate dehydrogenase *in vitro*	*P. triangulata*	44
59	Aztequynol A	—	*Petrosia* sp.	45
60	Aztequynol B	—	*Petrosia* sp.	45
61	Osirisyne A	—	*Haliclona osiris*	46
62	Osirisyne B	—	*H. osiris*	46
63	Osirisyne C	—	*H. osiris*	46
64	Osirisyne D	—	*H. osiris*	46
65	Osirisyne E	—	*H. osiris*	46
66	Osirisyne F	—	*H. osiris*	46
67	Aikupikanyne F	—	*Callyspongia* sp.	20
68	Haliclonyne	—	*Haliclona* sp.	47
69	Callyspongynic acid	α-glucosidase inhibitor	*P. corticata*	41, 48 and 49
70	Corticatic acid D	Geranylgeranyltransferase type I inhibitor	*P. corticata*	41, 48 and 49
71	Corticatic acid E		*P. corticata*	41, 48 and 49
72	(5*Z*,9*Z*)-22-Methyl-5,9-tetracosadienoic acid	Cytotoxic activity against mouse Ehrlich carcinoma cells and a hemolytic effect on mouse erythrocytes	*Stelletta* sp.	50
73	Stellettic acid C	Exhibited marginal to moderate toxicity to five human tumour cell lines	*Stelletta* sp.	51
74	Not given	Cytotoxic to human leukemia cells	*Stelletta* sp.	52
75	Petroformynic acid B	Cytotoxic	*Petrosia*	53
76	Petroformynic acid C		*Petrosia*	53
77	Heterofibrin A_1_	Inhibited lipid droplet formation	*Spongia* sp.	54
78	Officinoic acid B	—	*Spongia officinalis*	55
79	Fulvyne A	Against a chloramphenicol-resistant strain of *Bacillus subtilis*	*Haliclona fulva*	56
80	Fulvyne B		*H. fulva*	56
81	Fulvyne C		*H. fulva*	56
82	Fulvyne D		*H. fulva*	56
83	Fulvyne E		*H. fulva*	56
84	Fulvyne F		*H. fulva*	56
85	Fulvyne G		*H. fulva*	56
86	Fulvyne H		*H. fulva*	56
87	Fulvyne I		*H. fulva*	56
88	Petrosynic acid A	—	*Petrosia* sp.	57
89	Petrosynic acid B	—	*Petrosia* sp.	57
90	Petrosynic acid C	—	*Petrosia* sp.	57
91	Petrosynic acid D	—	*Petrosia* sp.	57

**Table 4:** Branched chain polyunsaturated fatty acids from algae

Number	Names	Bioactivities	Sources	Reference(s)
92	(10*E*,15*Z*)-(9*S*,12*R*,13*S*)-9,12,13-Trihydroxyoctadeca-10,14-dienoicacid	—	*Lyngbya majuscula*	58
93	(5*Z*,8*E*,10*E*)-11-Fomylundeca-5,8,10-trienoic acid	Antimicrobial	*Laurencia hybrida*	59
94	(2*Z*,5*Z*,7*E*,11*Z*,14*Z*)-9-Hydroxyeicosa-2,5,7,11,14-pentaenoic acid	Antimicrobial	L. *hybrida*	59
95	Acyclicditerpene	—	*Bifurcaria bifurcate*	60
96	Ptilodene	Inhibited both 5-lipoxygenase and Na^+^/K^+^A TPase	*Ptilota filicina*	61
97	12-(*S*)-Hydroxyeicosapentaenoic acid	Inhibitor of platelet aggregation	*Murrayella periclados*	62
98	9-Hydroxypentaenoic acid	—	*Laurencia hybrid*	63
99	Turbinaric acid	Cytotoxic	*Turbinaria ornata*	64
100	(12*R*,13*R*)-Dihydroxyeicosa-5(*Z*),8(*Z*),10(*E*),14(*Z*)-tetraeonic acid	Modulated fMLP-induced superoxide anion generation in human neutrophils; inhibited the conversion of arachidonic acid to lipoxygenase products by human neutrophils; inhibited the functioning of the dog kidney Na^+^/K^+^ ATPase	*Farlowia mollis*	65
101	(12*R*,13*R*)-Dihydroxyeicosa-5(*Z*),8(*E*),10(*E*),14(*Z*),17(*Z*)-pentaenoic acid		*F. mollis*	65
102	(10*R*,11*R*)-Dihydroxyoctadeca-6(*Z*),8(*E*),12(*Z*)-trienoic acid		*F. mollis*	65
103	(5*Z*,8*Z*,10*E*,12*R*,13*R*,14*Z*)-12,13-Dihydroxyeicosa,5,8,10,14-tetraenoic acid	—	*F. mollis*	65
104	(5Z,8Z,10E,12R,13S,14Z)-12,13-dihydroxyeicosa-5,8,10,14-tetraenoic acid	—	*F. mollis*	66
105	(6*Z*,9*E*,11*E*,13*E*)-9-Formyl-15-hydroxyheptadeca-6,9,11,13-tetraenoic acid	—	*Acrosiphonia coalita*	67
106	(9*E*,11*E*,13*E*)-9-Formyl-15-hydroxyheptadeca-9,11,13-trienoic acid	—	*A. coalita*	67
107	(6Z,9E,11E,13E)-9-formyl- 15-oxoheptadeca-6,9,11,13-tetraenoic acid	—	*A. coalita*	67
108	(10*E*,12*Z*,14*E*)-16-Hydroxy-9-oxooctadeca-10,12,14-trienoic acid	—	*A. coalita*	67
109	(10*E*,12*E*,14*E*)-16-hydroxy-9-oxooctadeca-10,12,14-trienoic acid	—	*A. coalita*	67
110	(9*Z*,11*R*,12*S*,13*S*,152)-12,13-Epoxy-11-hydroxyoctadeca-9,15-dienoic acid	—	*A. coalita*	67
111	(9*Z*,11*R*,12*S*,13*S*)-12,13-Epoxy-11-hydroxyoctadeca-9-enoic acid	—	*A. coalita*	67
112	(9*R*,10*R*,11*S*,12*Z*,152)-9,10-Epoxy-11-hydroxyoctadeca-12,15-dienoic acid	—	*A. coalita*	67
113	(9*R*,10*R*,11*S*,122)-9,10-Epoxy-11-hydroxyoctadeca- 12-enoic acid	—	*A. coalita*	67
114	Not given	—	*Laminaria sincluirii*	68
115	Not given	—	*L. sincluirii*	68
116	9,11-Dodecadienoic acid	—	*L. sincluirii*	68
117	(13*R*)-13-hydroxyarachidonic acid	—	*Lithothamnion coralloides*	69 and 70
118	(12*S*)-12-Hydroxyeicosatetraenoic acid	—	*M. periclados*	71
119	(6*E*)-Leukotriene B_4_	—	*M. periclados*	71
120	Hepoxilin B_3_	—	*M. periclados*	71
121	Hepoxilin B_3_	—	*M. periclados*	71
122	Hepoxilin B_4_	—	*M. periclados*	71
123	Hepoxilin B_4_	—	*M. periclados*	71
124	(5*R*,6*S*,7*E*,9*E*,11*Z*,14*Z*)-5,6-Dihydroxyicosa-7,9,11,14-tetraenoic acid	—	*Rhodymenia pertusa*	72
125	(5R*,6S*,7*E*,9*E*,11*Z*,14*Z*,17*Z*)-5,6-Dihydroxyicosa-7,9,11,14,17-pentaenoic acid	—	*R. pertusa*	72
126	(6*E*,8*Z*,11*Z*,14*Z*)-5-Hydroxyicosa-6,8,11,14-tetraenoic acid	—	*R. pertusa*	72
127	(6*E*,8*Z*,11*Z*,14*Z*,17*Z*)-5-Hydroxyicosa-6,8,11,14,17-Pentaenoic acid	—	*R. pertusa*	72
128	8,12-Octadecadienoic acid	—	*Corallina officinalis*	73
129	(8*E*,12*Z*,15*Z*)-10-Hydroxy-8,12,15-trien-4,6-diynoic acid	—	*Caulerpa racemosa*	74

**Table 5:** Branched chain polyunsaturated fatty acids from Coelenterate, Marine fungus, Arthropoda, Bacterium

Number	Names	Bioactivities	Sources	Reference(s)
130	Leiopathic acid	—	*Leiopathes* sp.	75
131	5,9,11,14,17-Eicosapentaenoic acid	—	*Leiopathes* sp.	75
132	5,9,11,14,17-Eicosapentaenoic acid	—	*Leiopathes* sp.	75
133	(11*R*)-Hydroxyeicosatetraenoic acid	—	*Plexaurella dichotoma*	76
134	(5*Z*,9*Z*)-14-methylpentadeca-5,9-dienoic acid	Inhibited the growth of Gram positive bacteria	*Eunicea succinea*	77
135	6,9,12,16,18-Tetracosapentaenoic acid	Inhibited tube-formation in a human endothelial cell line model of angiogenesis	*Sinularia numerosa*	78
136	Dendryphiellic acid A	—	*Dendryphiella salina*	79 and 80
137	Dendryphiellic acid B	—	*D. salina*	79 and 80
138	Curvulalic acid	—	*Curvularia* sp.	81
139	2,4-Decadienoic acid	—	*Xylaria* sp.	82
140	(5Z,8*R*,9E,11*Z*,14Z,17Z)-8-hydroxyeicosa-5,9,11,14,17-pentaenoic acid	—	*Balanus balanoides*, *Eliminus modestus*	83
141	8,13-Dihydroxyeicosapentaenoic acid	A muscle stimulatory factor in the barnacle *Balanus balanus*	*Balanus balanus*	84
142	(9Z,12Z)-7-hydroxyoctadeca-9,12-dien-5-ynoic acid	Ichthyotoxic	*L. farinosa*	33
143	Macrolactic acid	—	Unidentified Gram-positive bacterium	85
144	Isomacrolactic acid	—	Unidentified Gram-positive bacterium	85
145	Ieodomycin C	Antimicrobial	*Bacillus* sp.	86
146	Ieodomycin D		*Bacillus* sp.	86
147	Linieodolide B	Antibacterial; antifungal	*Bacillus* sp.	87 and 88

#### 3.2.1 Sponges

Acetylenic acids, 39–42, identified as the corresponding ethyl esters, were obtained from a specimen of *Phakellia carduus* obtained from a depth of 350 m by trawling.^21^ Studies on the biosynthesis of the branched fatty acids 43 and 44 (from *Jaspis stellifera*) indicated that the unusual long-chain fatty acids were formed by elongation of shorter branched fatty acids, and that methyl branching did not occur after elongation of the chain.^35^ An unusually short fatty acid, (5*Z*,9*Z*)-hexadeca-5,9-dienoic acid 45, was obtained from *Chondrilla nucula*.^36^ Relatively large amounts of the eicosanoids 46 and 47 and hydroxy acids 48 and 49 were found in *Echinochalina mollis* from the Coral Sea; they were isolated as the corresponding methyl esters and identified by interpretation of spectral data.^37^ A stereoselective route to the methyl branched (5*Z*,9*Z*)-eicosa-5,9-dienoic acids 50 and 51 found in *Erylus forrnosus*^38^ has been described.^39^ Petrosolic acid 52 that inhibited HIV reverse transcriptase was the constituent of a Red Sea *Petrosia* sp.^40^ Corticatic acids A–C 53–55 are antifungal acetylenicacids from *Petrosia corticata* from Japanese waters.^41^ Spectroscopic analysis had resulted in a tentative structure for nepheliosyne A 56 from an Okinawan sponge of the genus *Xestospon*.^42^*Pellina triangulata* from Truk in Micronesia contained triangulynic acid 57, which is a cytotoxic polyacetylene that was most active against leukemia and colon tumour lines.^43^ Pellynic acid 58, which inhibited inosine monophosphate dehydrogenase *in vitro*, was obtained from *Pellina triangulata* from Chuuk (Truk) Atoll.^44^ Aztequynols A 59 and B 60 were C-branched acetylenes from a *Petrosia* sp. from New Caledonia.^45^ A more complex series of highly oxygenated C47 polyacetylenes, osirisynes A–F 61–66, were isolated as cytotoxins from a Korean specimen of *Haliclona osiris*.^46^ One polyacetylene, aikupikanynes F 67 was obtained from a *Callyspongia* sp. from the Red Sea.^20^ The polyacetylene carboxylic acid haliclonyne 68 was obtained from a *Haliclona* sp. from the Red Sea.^47^ Japanese specimens of *Callyspongia truncata* yielded the α-glucosidase inhibitor callyspongynic acid 69^48^ while corticatic acids D 70 and E 71^41^ were isolated from a Japanese *Petrosia corticata* and were found to be geranylgeranyltransferase type I inhibitors.^49^

A cytotoxic fatty acid, (5*Z*,9*Z*)-22-methyl-5,9-tetracosadienoic acid 72 was isolated from *Geodinella robusta* collected from the Sea of Okhotsk, Russia.^50^ An undescribed Korean species of *Stelletta* was found to contain a cytotoxic acetylenic acid: stellettic acid C 73 that exhibited marginal to moderate toxicity to five human tumour cell lines.^51^ From a seemingly identical *Stelletta* species, collected at a different Korean location, a desmethoxy analogue 74, was isolated; it was mildly cytotoxic to human leukemia cells.^52^ The cytotoxic petroformynic acids B 75 and C 76 were obtained from a *Petrosia* species (Katsuo-jim Is., Wakayama Pref., Japan).^53^ One acetylenic compound heterofibrin A_1_77 was isolated from a *Spongia* (Heterofibria) sp. collected by dredging in the Great Australian Bight. Heterofibrin A_1_ inhibited lipid droplet formation at 10 mM yet was not cytotoxic at similar concentrations.^54^ Officinoic acid B 78 is linear diterpene from *Spongia officinalis* (off Mazara del Vallo, Sicily).^55^ An extract of *Haliclona fulva* (Procida Is., Gulf of Naples, Italy) contained the nine acetylenes fulvyne A–I 79–87.^56^ Petrosynic acids A–D 88–91 (*Petrosia* sp., Tutuila, American Samoa) all displayed similar activity *versus* various HTCLs and non-proliferative human fibroblasts and hence no therapeutic window is available.^57^

#### 3.2.2 Algae

Malyngic acid 92 is not the acid that is associated with the malyngamides, but it has been shown to be (10*E*,15*Z*)-(9*S*,12*R*,13*S*)-9,12,13-trihydroxyoctadeca-10,14-dienoicacid.^58^ Unlike most metabolites from *Lyngbya majuscula*, malyngic acid was found in both shallow- and deep-water varieties. Research on *Laurencia hybrida* indicated that these lipid pools might contain undescribed bioactive metabolites. The antimicrobial constituents (5*Z*,8*E*,10*E*)-11-fomylundeca-5,8,10-trienoic acid 93 and (2*Z*,5*Z*,7*E*,11*Z*,14*Z*)-9-hydroxyeicosa-2,5,7,11,14-pentaenoic acid 94 might be considered as primary metabolites were it not for their bioactivity.^59^ The additional acyclicditerpene 95 has been reported from *Bifurcaria bifurcate*.^60^ Ptilodene 96 is an eicosanoid from *Ptilota filicina* that inhibited both 5-lipoxygenase and Na^+^/K^+^ ATPase.^61^ 12-(*S*)-Hydroxyeicosapentaenoic acid 97, which is a potent inhibitor of platelet aggregation, has been isolated in large quantities from *Murrayella periclados* and has been recognized as the compound previously identified^62^ as 9-hydroxypentaenoic acid 98 from *Laurencia hybrid*.^63^ The structure of turbinaric acid 99, which is a cytotoxic constituent of *Turbinaria ornata*, was elucidated from spectral data and confirmed by synthesis.^64^ A notable exception was the report of three biologically active eicosanoids, (12*R*,13*R*)-dihydroxyeicosa-5(*Z*),8(*Z*),10(*E*),14(*Z*)-tetraeonic acid 100, (12*R*,13*R*)-dihydroxyeicosa-5(*Z*),8(*E*),10(*E*),14(*Z*),17(*Z*)-pentaenoic acid 101, and (10*R*,11*R*)-dihydroxyoctadeca-6(*Z*),8(*E*),12(*Z*)-trienoic acid 102 that were isolated from the temperate red alga *Farlowia mollis*.^65^ The structure of a dihydroxy eicosanoid isolated from the red alga *Farlowia mollis* has been revised from (5*Z*,8*Z*,10*E*,12*R*,13*R*,14*Z*)-12,13-dihydroxyeicosa,5,8,10,14-tetraenoic acid 103^65^ to (5*Z*,8*Z*,10*E*,12*R*,13*S*,14*Z*)-12,13-dihydroxyeicosa-5,8,10,14-tetraenoic acid 104 as a result of the synthesis of the both *threo* and *erythro* isomers.^66^

The green alga *Acrosiphonia coalita* contains the oxylipins coalital, which may be an artefact caused by photoisomerization of the natural product, racemic (6*Z*,9*E*,11*E*,13*E*)-9-formyl-15-hydroxyheptadeca-6,9,11,13-tetraenoic acid 105, (9*E*,11*E*,13*E*)-9-formyl-15-hydroxyheptadeca-9,11,13-trienoic acid 106, (6*Z*,9*E*,11*E*,13*E*)-9-formyl-15-oxoheptadeca-6,9,11,13-tetraenoic acid 107, (10*E*,12*Z*,14*E*)-16-hydroxy-9-oxooctadeca-10,12,14-trienoic acid 108, (10*E*,12*E*,14*E*)-16-hydroxy-9-oxooctadeca-10,12,14-trienoic acid 109, (9*Z*,11*R*,12*S*,13*S*,152)-12,13-epoxy-11-hydroxyoctadeca-9,15-dienoic acid 110, (9*Z*,11*R*,12*S*,13*S*)-12,13-epoxy-11-hydroxyoctadeca-9-enoic acid 111, (9*R*,10*R*,11*S*,12*Z*,152)-9,10-epoxy-11-hydroxyoctadeca-12,15-dienoic acid 112, and (9*R*,10*R*,11*S*,122)-9,10-epoxy-11-hydroxyoctadeca-12-enoic acid 113, the acids all being isolated as the corresponding methyl esters.^67^ Three divinyl ethers, 114–116, were isolated along with a number of hydroxylated fatty acids from the Oregon brown alga *Laminaria sincluirii* and were identified by interpretation of spectral evidence.^68^ The absolute stereochemistry of (13*R*)-13-hydroxyarachidonic acid 117, which is a known eicosanoid from *Lithothamnion coralloides*,^69^ was determined by degradation and its biosynthesis from arachidonic acid was studied.^70^

The Caribbean alga *Murrayella periclados* contains a number of eicosanoids that include (12*S*)-12-hydroxyeicosatetraenoic acid 118, (6*E*)-leukotriene B_4_, 119 and *erythro* and *threo* isomers of hepoxilins B_3_, 120/121 and B_4_, 122/123.^71^ Four oxylipins (5*R*,6*S*,7*E*,9*E*,11Z,14*Z*)-5,6-dihydroxyicosa-7,9,11,14-tetraenoic acid 124, (5R*,6S*,7*E*,9*E*,11*Z*,14*Z*,17*Z*)-5,6-dihydroxyicosa-7,9,11,14,17-pentaenoic acid 125, (6*E*,8*Z*,11*Z*,14*Z*)-5-hydroxyicosa-6,8,11,14-tetraenoic acid 126, and (6*E*,8*Z*,11*Z*,14*Z*,17*Z*)-5-hydroxyicosa-6,8,11,14,17-pentaenoic acid 127 were isolated from *Rhodymenia pertusa*.^72^ An oxylipin 128 was obtained from *Aspergillus flavus*, (red alga *Corallina officinalis*, Yantai, China).^73^ Studies on a *Caulerpa racemosa* (Zhanjiang coastline, China) led to the isolation of the acetylenic fatty acid (8*E*,12*Z*,15*Z*)-10-hydroxy-8,12,15-trien-4,6-diynoic acid 129.^74^

#### 3.2.3 Coelenterate

Leiopathic acid 130 and two known eicosanoids, 131 and 132, were isolated from a black coral, *Leiopathes* sp., collected at St Paul Island in the South India Ocean.^75^. (11*R*)-Hydroxyeicosatetraenoic acid 133, a proposed intermediate on the pathway to prostanoids in coelenterates, has been found in the gorgonian *Plexaurella dichotoma*.^76^ The gorgonian *Eunicea succinea* contained (5*Z*,9*Z*)-14-methylpentadeca-5,9-dienoic acid 134, which inhibited the growth of Gram positive bacteria.^77^ Oxylipin 135, isolated by bioassay-directed fractionation (*Sinularia numerosa*, Kagoshima Prefecture, Japan), inhibited tube-formation in a human endothelial cell line model of angiogenesis.^78^

#### 3.2.4 Marine fungus

The marine deuteromycete *Dendryphiella salina* produced an unusual group of trinor-eremophilane and eremophilane derivatives.^79^ The structures of dendryphiellic acids A 136 and B 137 were proposed on the basis of spectral and chemical studies as well as comparison of their spectral data with those of dendryphiellin A.^80^ A *Curvularia* sp. (sea fan *Annella* species, Similan Islands, Phangnga, Thailand) yielded the metabolites curvulalic acid 138.^81^ The lipid 139 was obtained from *Xylaria* sp.^82^

#### 3.2.5 Arthropoda

The structure of the hatching factor of the barnacles *Balanus balanoides* and *Eliminus modestus* has been confirmed by synthesis to be (5*Z*,8*R*,9*E*,11*Z*,14*Z*,17*Z*)-8-hydroxyeicosa-5,9,11,14,17-pentaenoic acid 140.^83^ 8,13-Dihydroxyeicosapentaenoic acid 141 was identified as a muscle stimulatory factor in the barnacle *Balanus balanus*.^84^

#### 3.2.6 Bacterium

The metabolite, (9*Z*,12*Z*)-7-hydroxyoctadeca-9,12-dien-5-ynoic acid 142, was ichthyotoxic.^33^ An unidentified Gram-positive bacterium from a deep-sea sediment core produced macrolactic acid 143 and isomacrolactic acid 144.^85^ The fatty acids, ieodomycins C 145 and D 146 from *Bacillus* sp. (sediment, Ieodo, South Korea) had broad spectrum antimicrobial activity.^86^*Bacillus* sp. (sediment, Ieodo Reef, S. Korea)^87^ produced the unsaturated fatty acid linieodolide B 147, with modest antibacterial and antifungal activity.^88^

## 4 Biosynthetic pathways

**Fig. 6:**
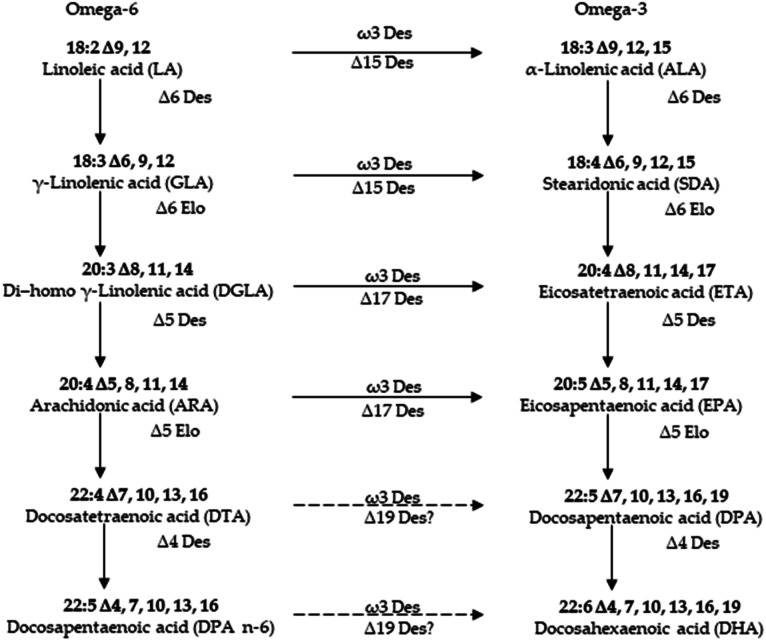
Pathway for the biosynthesis of long chain polyunsaturated fatty acids in microalgae.

PUFAs are gaining importance due to their innumerable health benefits. The most common source of PUFAs is of marine origin. Hence, understanding their biosynthesis in marine origin has attained prominence in recent years.^89,90^ Rabbitfish *Siganus canaliculatus* was the first marine teleost demonstrated to have the ability to biosynthesize C20–22 long-chain polyunsaturated fatty acid (LC-PUFA) from C18 PUFA precursors, which is generally absent or low in marine teleosts.^91^ The marine diatom *Phaeodactylum tricornutum* accumulates eicosapentaenoic acid (EPA, 20:5n-3) as its major component of fatty acids. To improve the EPA production, delta 5 desaturase, which plays a role in EPA biosynthetic pathway, was characterized in marine diatom *Phaeodactylum tricornutum*.^90^ There is currently considerable interest in understanding how the biosynthetic pathways of highly unsaturated fatty acids (HUFA) are regulated in fish. The aim is to know if it is possible to replace fish oils (FO), rich in HUFA, by vegetable oils (VO), poor in HUFA and rich in their 18 carbon fatty acid precursors, in the feed of cultured fish species of commercial importance.^92^ Although many better insights into the synthesis of eicosapentaenoic acid (EPA) and docosahexaenoic acid (DHA) in marine microalgae,^93^ there are still a little known about biosynthetic processes of most isolated UFAs of marine resources.^70,94^ Thus, more investigation should be carried out for these marine derived UFAs in the coming researches (Fig. 6).

## 5 Beneficial application

It is well-known that polyunsaturated fatty acids n-3 (PUFAn-3) are very important for human health and nutrition.^1^ As an example, highly unsaturated long-chain omega-3 fatty acids, derived from the liver of white lean fish, flesh of fatty fish, and blubber of marine mammals, exhibit important biological activities.^95^ They also serve as the building block fatty acids in the brain, retina, and other organs with electrical activity. Hence, inclusion of oils containing docosahexaenoic acid (DHA) in the diet of pregnant and lactating women as well as infants is encouraged.^95^

**Fig. 7:**
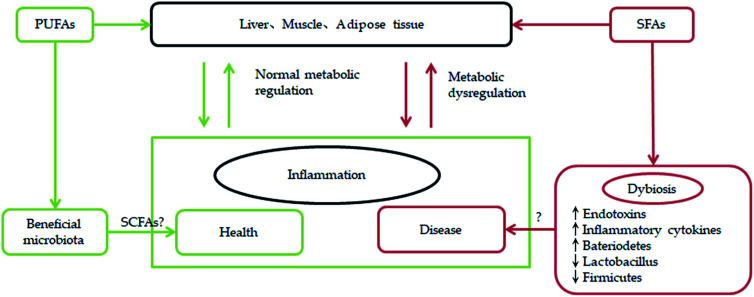
Impact of SFA and PUFA on gut microbiota and metabolic regulation.

In addition, some polyunsaturated fatty acids from marine microalgae are found to modulate lipid metabolism disorders and gut microbiota.^96^ According to the survey results, high saturated fatty acid and high monounsaturated fatty acid diets have an adverse effect on the gut microbiota and high saturated fatty acids are associated with unhealthy metabolic status, while polyunsaturated fatty acid does not have a negative impact on gut microbiota.^97^ Through previous studies we find that connecting with gut microbiota, PUFAs can be more beneficial for human health. For example, increasing anti-obesogenic microbial species in the gut microbiota population by appropriate n-3 PUFAs can be an effective way to control or prevent metabolic diseases.^98^ Furthermore, a link has been established between n-3 PUFAs and gut microbiota especially with respect to inflammation (Fig. 7). A few related researchs show that after omega-3 PUFA supplementation, *Faecalibacterium*, often associated with an increase in the Bacteroidetes and butyrate-producing bacteria belonging to the Lachnospiraceae family, has decreased. Omega-3 PUFAs perform a positive action on diseases by reverting the microbiota composition and increasing the production of anti-inflammatory compounds like short-chain fatty acids.^99^ According to the link between n-3 PUFAs and gut microbiota, which is associated with inflammation, some scholars proposing that an optimal level of LC-PUFAs nurtures the suitable gut microbiota that will prevent dysbiosis. The synergy between optimal LC-PUFAs and gut microbiota helps the immune system overcome the immunosuppressive tumour microenvironment.^100^

Although many scholars have devoted themselves to the study of polyunsaturated fatty acids, they are limited to the more famous unsaturated fatty acids. There is still lack of investigation of the beneficial application of these polyunsaturated fatty acid derivatives with similar structural characteristics. Thus, more investigation should focus on fatty acid physiological roles and applications in human health and disease and the interaction with gut microbiota.^101^

## 6 Conclusions

**Fig. 8:**
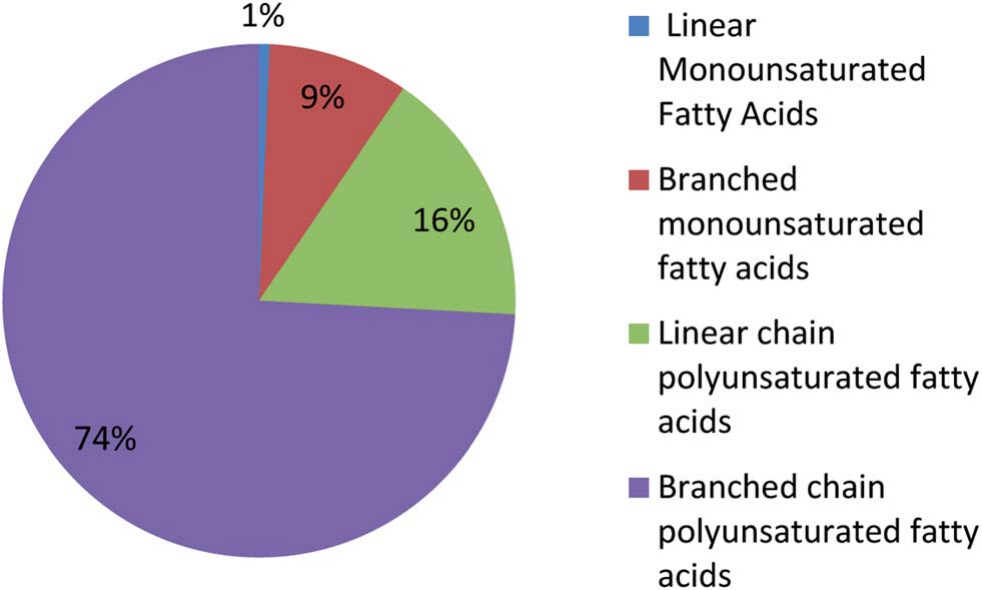
The distribution of UFAs reported from marine organisms.

**Fig. 9:**
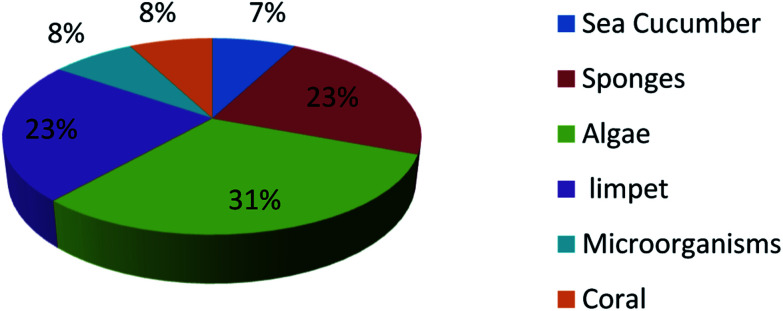
Origin of branched monounsaturated fatty acids.

**Fig. 10:**
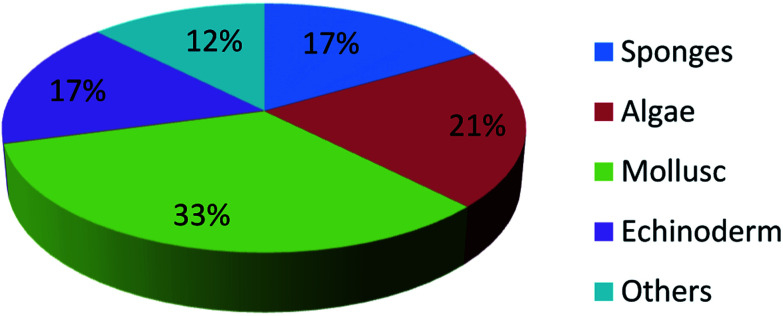
Origin of linear chain polyunsaturated fatty acids.

**Fig. 11:**
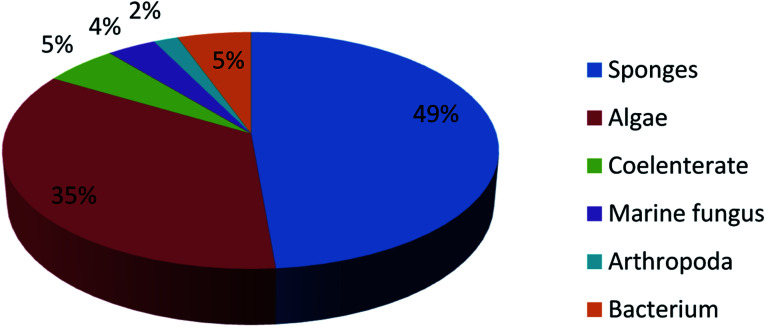
Origin of branched chain polyunsaturated fatty acids.

UFAs are ubiquitous in many marine organisms.^3,102,103^ Although these UFA secondary metabolites have been obtained since the early 20th century, they only recently draw significant interests because of the diverse range of their biological and nutritional properties.^104^ However, there is still lack of a comprehensive review about the structural characterizations, biological and nutritional properties, proposed biosynthetic processes, and beneficial application of marine derived UFAs. 1978 to 2018, the main structural types of UFAs obtained from marine organisms is branched chain PUFAs, accounting for 74% of the total (Fig. 8), the main natural source of branched monounsaturated fatty acids isolated from marine organisms is coral, accounting for 31% (Fig. 9), while linear chain polyunsaturated fatty acids obtained from marine organisms is mollusc, accounting for 33% (Fig. 10), the preponderant natural marine source of PUPAs is arthropoda, accounting for 49% (Fig. 11). Although omega-3 fatty acid,^6^ eicosapentaenoic acid (EPA) and docosahexaenoic acid (DHA) from marine organisms, have dramatically increased as excellent sources of nutrients, it is indicated that the biological activities of most of the UPAs are not investigated (Tables 1–3), and the little known about the biosynthetic pathways of these isolated UPAs. In addition, there is no report about new UFAs isolated from marine resources during 2016 to 2018. Thus, the further investigation of marine derived PUPAs should focus on their and beneficial application mediated by gut microbiota.

## Conflicts of interest

The authors declare no conflict of interest.

## Supplementary Material
